# Identification of characteristic aroma compounds in raw and thermally processed African giant snail (*Achatina fulica*)

**DOI:** 10.1186/s13065-018-0413-6

**Published:** 2018-04-24

**Authors:** Ola Lasekan, Megala Muniady, Mee Lin, Fatma Dabaj

**Affiliations:** Department of Food Technology, University Putra Malaysia, UPM, 43400 Serdang, Malaysia

**Keywords:** African giant snails, Aroma compounds, Thermal process, AEDA, OAVs

## Abstract

**Background:**

Food flavor appreciation is one of the first signals along with food appearance and texture encountered by consumers during eating of food. Also, it is well known that flavor can strongly influence consumer’s acceptability judgment. The increase in the consumption of snail meat across the world calls for the need to research into the aroma compounds responsible for the distinctive aroma notes of processed snail meat.

**Results:**

The odorants responsible for the unique aroma notes in thermally processed giant African snail meats were evaluated by means of aroma extract dilution analysis (AEDA), gas chromatography–olfactometry (GC–O) and odor activity values (OAVs) respectively. Results revealed significant differences in the aroma profiles of the raw and thermally processed snail meats. Whilst the aroma profile of the raw snail meat was dominated with the floral-like β-ionone and β-iso-methyl ionone, sweaty/cheesy-like butanoic acid, and the mushroom-like 1-octen-3-one, the boiled and fried samples were dominated with the thermally generated odorants like 2-methylpyrazine, 2,5-dimethylpyrazine, 2-acetylthiazole and 2-acetylpyridine.

**Conclusion:**

Finally, results have shown that sotolon, 2-acetyl-1-pyrroline, 2-furanmethanethiol, 2-methylbutanal, 1-octen-3-one, octanal, furanone, 2-methoxyphenol, 2-acetylpyridine, 2-acetylthiazole, and 2-methylpyrazine contributed to the overall aroma of the thermally processed snail meat.

## Background

The giant African snail (*Achatina fulica* Bowdich) belongs to the Achatinoidea family and its native to East Africa. However, it has been widely distributed to different parts of the world such as; China [1], Taiwan [2], India, West Indies and the United States [3]. The snail’s habitat covers the dense tropical forest of West Africa, Pacific Islands, Southern and Eastern Asia, and the Caribbean [4]. Different breeds of land snails have been reported and the most common breeds in Africa are *Achatina achatina, Achatina fulica, Achachatina marginata* and *Limocolaria* species [5]. The giant African snail is considered as one of the worst invasive species, because of its impact on agricultural and horticultural crops [6].

In spite of its invasive activities, African giant snails have been reported to exhibit antimicrobial properties. For instance, snails produce mucin in abundance in their mucus secretion. The mucin also called slim contains a bactericidal glycoprotein known as ‘achacin’ [7]. Also, the use of snail mucin for wound healing has been reported [8]. The giant African snails are highly relished delicacy in some parts of Africa, Taiwan, and South Korea [9]. France is the world leading consumer of snails followed, in order by Italy, Spain and Germany [10]. The snails are excellent sources of nutrition, as they contain abundant levels of calcium, phosphorous, magnesium and protein [11]. In addition, the distinctive aroma of fried snails is very effective in enhancing the flavor of dishes.

Several studies have been reported on the volatile composition of edible freshwater mollusks such as clams [12], mussels, shrimp and squid [13]. Sekiwa et al. [12] identified 49 compounds in clams among which were; 2,5-dimethyl-4-hydroxy-3(2H)-furanone, 2-acetyl-2-thiazoline, 2-acetylthiazole and 3-methylthiopropanal. Whereas, Giogios et al. [13], reported high amounts of aldehydes, furans, and N-containing compounds (i.e. pyridine, pyrazines and pyrroline) in mussels. However, for the overall aroma of oysters, the main compounds were 3-cyclohexene-1-ethanol (*Z*)-1,5-octadien-3-ol, 2-octen-1-ol, benzaldehyde and lilac aldehyde [14]. In another study, on the potent aroma compounds in dried scallops (*Patinopecten yessoensis*), Chung, Yung, Ma and Kin [15] found pentanal, 2-methylene-hexanal, 1,2-dichlorobenzene, 1-methoxy-4-(2-propenyl)-benzene, ethyl benzoate and (*Z*)-jasmone as some of the potent compounds. The effects of thermal processing and/or conservation treatments on volatile compounds generation in fish and fish products have also been documented. For example, while hexanal, 2-ethyl-1-hexanol, dimethylsulphide, 6-methyl-5-hepten-2-one, nonanal, 1-octen-3-one and y-butyrolactone were reported as the major volatile compounds in raw Mediterranean shrimps [16], the cooked shrimps produced appreciable amounts of 2-methylbutanal, 3-methylbutanal. 2,6-dimethylpyrazine, dimethylsufoxide, 1-dodecanol in addition to hexanal and dimethyl sulphide [16]. Li et al. [17] reported significant amounts of furans in fried grass fillet carp (*Ctenopharyngodon idellus*). The major compounds identified in the fried fillets were: 6-heptyltetrahydro-2H-pyran-2-one, 2,5-dimethyl-4-hydroxy-3(2H)-furanone, 5-hydroxymethylfurfural, decanal, 3-methyl-1-butanol, 2-pentylfuran and 2,5-dimethyl-3-ethylpyrazine. Apart from the effect of thermal processing, conservation treatment such as salting has been known to influence volatile production. For instance, Conte et al. [18] reported that salted red mullet (*Mullus surmuletus*) exhibited high levels of hexanal, heptanal and (Z)-4-heptanal.

From a consumer perspective, the most appealing features of a processed snail meat are its flavor and nutrition. Food flavor appreciation is one of the first evaluation signals along with food appearance and texture encountered by consumers during eating [19].

However, to the best of our knowledge, there has been no report on the odorants responsible for the typical flavor of processed giant African snail. The aim of this study was to evaluate the potent aroma-active compounds in thermally processed giant African snail.

## Results and discussion

### Odorants in raw snail meat

**Fig. 1:**
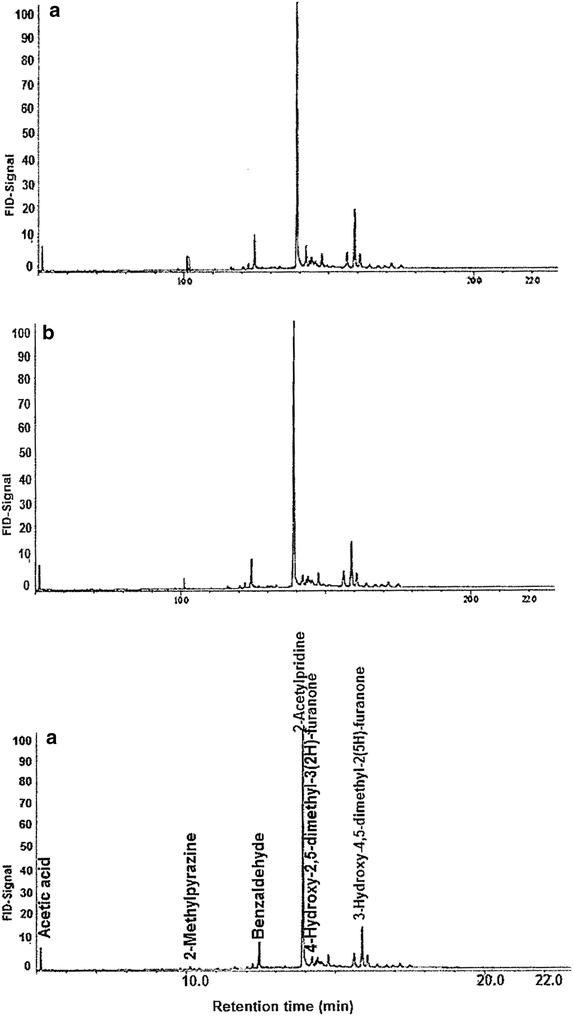
Characteristic gas chromatograms of solvent extracted African giant snail meat: **a** raw, **b** boiled and **c** fried

The aroma-active compounds in raw and thermally processed African giant snail meat (*A. fulica*) were evaluated. The most aroma-active components identified in the raw snail meat are listed in Table 1 and Fig. 1 respectively. The application of aroma extract dilution analysis (AEDA) and gas chromatography olfactometry (GC–O) revealed 13 odor-active compounds with FD factors from 4 to 32. Of this number, 8 odorants were obtained in the neutral basic fractions (NBF), while 5 odorants were found in the acidic fraction (AF). The major odorants with flavor dilution (FD ≥ 8) in the raw snail meat were 1-octen-3-one, benzaldehyde, octanal, β-ionone and β-iso-methyl ionone. Odorant with the least FD of 2 was identified as 2,3-pentanedione. 2,3-Pentanedione, 1-octen-3-one, benzaldehyde and octanal have been widely reported in different species of mollusks such as shellfish [20], squid [21] and steamed mangrove crab [22]. However, β-iso-methyl ionone (Apo-carotenoid) to the best of our knowledge has not previously been detected or described in snail meat or any other mollusks.

**Table 1:** Most aroma-active components (FD ≥ 4) in raw and boiled giant snail meat (*A. fulica*)

No	Compound^a^	Odour note	Fraction^b^	DB5	FFAP	FD boiled	FD raw
1	Acetoin	Buttery	NBF	nd	1275	4	4
2	Acetic acid	Vinegar-like	AF	635	1450	4	4
3	2-Methylbutanal	Malty	NBF	663	912	16	nd
4	2,3-Pentanedione	Caramel	NBF	696	1054	4	2
5	Butanoic acid	Sweaty, cheesy	AF	835	1619	4	4
6	2-Methylpyrazine	Popcorn	NBF	820	nd	8	nd
7	2,5-Dimethylpyrazine	Nutty, roasty	NBF	906	nd	8	nd
8	Benzaldehyde	Almond-like	NBF	963	1524	16	8
9	1-Octen-3-one	Mushroom	NBF	977	1295	32	32
10	Octanal	Citrus	NBF	1006	1276	16	8
11	2-Acetylthiazole	Roasty, earthy	NBF	1020	1624	64	nd
12	2-Acetylpyridine	Popcorn	NBF	1031	1551	128	nd
13	3-Hydroxy-4,5-dimethyl-2(5H) furanone (Sotolon)	Seasoning	AF	1107	2200	16	nd
14	β-ionone	Floral	NBF	1457	1959	4	8
15	β-iso-methyl ionone	Floral	NBF	1534	nd	4	8
16	Octadecanal	Fatty	NBF	1818	2179	8	4
17	Hexadecanoic acid	Waxy	AF	1984	2940	4	4
18	Octadecanoic acid	Mild fatty	AF	2178	nd	4	4
19	9,12-Octadecadienoic acid (*Z,Z*)	Faint fatty	AF	2183	nd	4	4

*AF* acidic fraction, *NBF* neutral and basic fraction, *FD* flavour dilution

^a^ Compounds were identified by comparing their retention indices on DB-5 and FFAP columns, mass spectra, and their aroma impressions were compared with the respective reference compounds

^b^ Fractions in which the odorants were detected by GC–O after fractionation

### Odorants in boiled snail meat

The aroma-active compounds in boiled African giant snail meat (*A. fulica*) were also evaluated by AEDA and GC–O respectively. A total of 19 odor-active compounds with flavor dilution (FD) factors ranging from 4 to 128 (Table 1) were detected. Of this number, 13 odorants were obtained in the neutral basic fractions (NBF), while the other 6 odorants were found in the acidic fractions (AF). The identified odorants produced an array of aroma nuances such as: buttery, malty, caramel-like, sweaty/cheesy, popcorn-like, mushroom, seasoning, floral and roasty. Furthermore, results of the AEDA revealed that 2-acetylpyridine, 2-acetylthiazole, 1-octen-3-one, benzaldehyde, 2-methylbutanal, octanal and 3-hydroxy-4,5-dimethyl-2(5H)-furanone (sotolon) possessed the highest FD factors (Table 1). Lower FD factors were produced by acetoin, 2-methylpyrazine, 2,5-dimethylpyrazine, octadecanal, acetic acid, 2,3-pentanedione, butanoic acid, β-ionone, β-iso-methyl ionone, hexadecanoic acid, octadecanoic acid and 9,12-octadecadienoic acid (*Z,Z*).

**Fig. 2:**
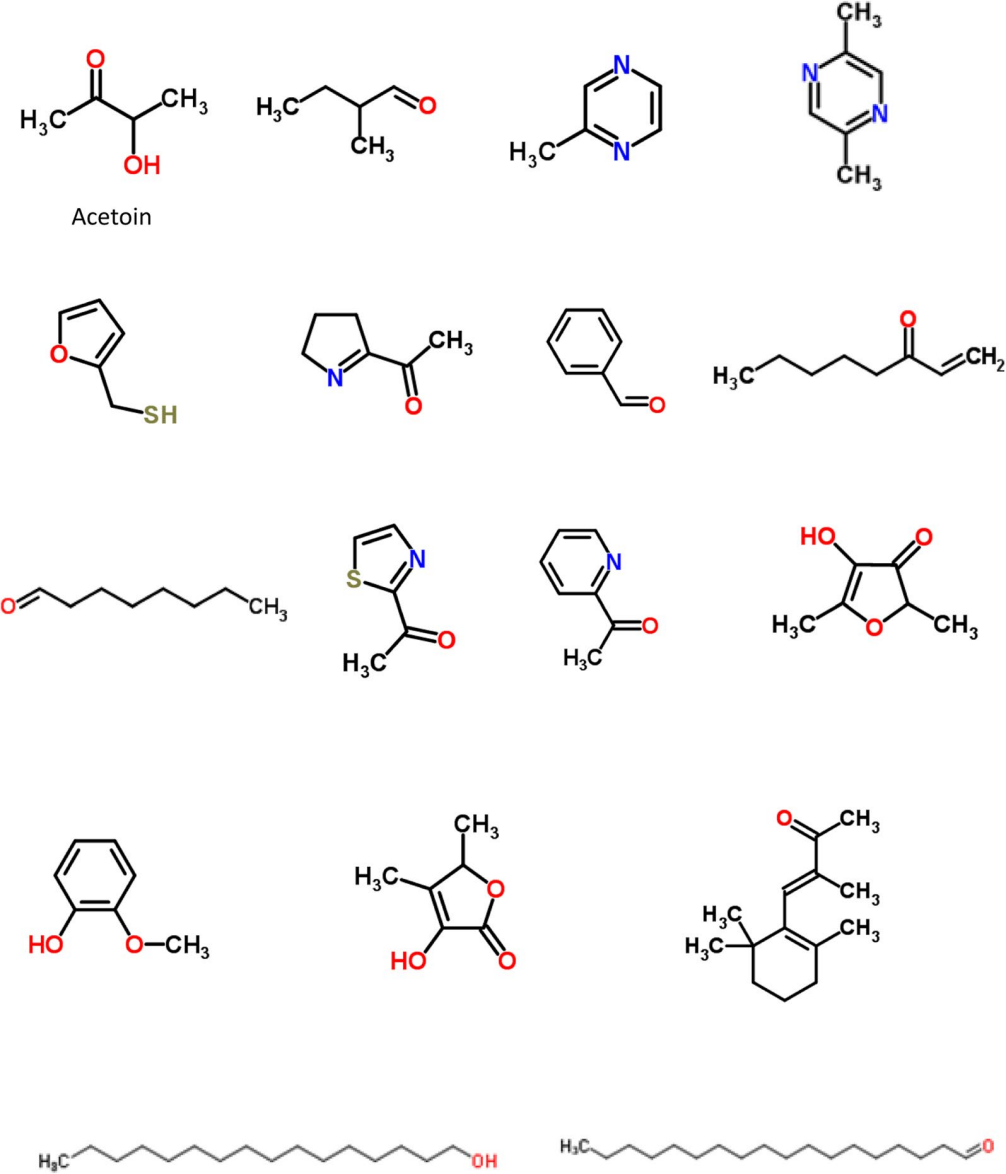
Aroma-active compounds in boiled and fried African giant snail meat

**Fig. 3:**
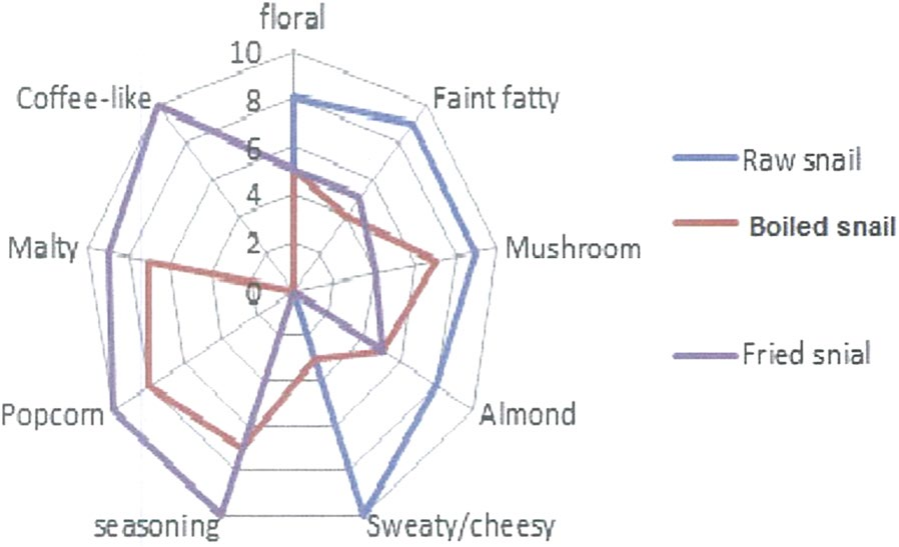
Comparative aroma profiles of raw, boiled and fried snail meat

A comparative analysis of the aroma profiles of raw and boiled snail meats revealed a significant number of thermally generated odorants in the boiled snails. Some of the identified odorants were; 2-methylpyrazine, 2,5-dimethylpyrazine, 2-acetylthiazole and 2-acetylpyridine (Fig. 2). Whereas, the aroma profile of the raw snail meat was dominated by floral, faint fatty, mushroom and sweaty/cheesy notes, the boiled snail meat elicited malty, popcorn-like, seasoning and mushroom nuances (Fig. 3). While the aroma notes developed in the boiled snail meat strongly increased in the fried snail samples, the faint fatty and mushroom notes decreased significantly. In order to elucidate the reasons behind this observation, the fried snail meats were subjected to AEDA and GC–O as earlier describe for the boiled snails.

### Odorants in fried snail meat

A total of 22 aroma-active compounds were detected with FD factors between 4 and 256 (Table 2). Of this number, 16 odorants were obtained in the NBF while the rest were acidic fractions. The aroma-active compound with the highest FD factor was the popcorn-like 2-acetyl-1-pyrroline. This was followed by the roasty/earthy 2-acetylthiazole (FD factor of 64), 2-furanmethanethiol with an FD factor of 32, 2-methoxyphenol with an FD factor of 32 and the seasoning-like 3-hydroxy-4,5-dimethyl-2(5H)-furanone with an FD factor of 32. Others with lower FD factors were; 2-methylbutanal, 2-methylpyrazine, benzaldehyde, 4-hydroxy-2,5-dimethyl-3(2H)-furanone, 2,5-dimethylpyrazine and 2-acetylpyridine. However, 2-furanmethanethiol, 4-hydroxy-2,5-dimethyl-3(2H)-furanone, 2-methoxyphenol, 2-acetyl-2-thiazoline and some saturated long chain aldehydes were detected only in fried snail and not in boiled snail. With the exception of the aforementioned compounds, the same sets of odorants identified in boiled snails were also detected in fried snails. Worthy of note is the significant presence of aroma compounds eliciting the popcorn-like note in the fried snail meat. 2-Acetyl-1-pyrroline, 2-acetylpyridine and 2-acetyl-2-thiazoline are examples of compounds with the popcorn-like note. 2-Acetyl-2-thiazoline which had the lowest FD factor (4) among the group has previously been identified as aroma component of cooked meat of spiny lobster [23] and American lobster (*Homarus americanus*) [24]. This aroma-active compound was shown to be thermally generated by the reaction of cysteine with ribose [25].

**Table 2:** Most aroma-active components (FD ≥ 4) in Fried giant snail meat (*A. fulica*)

No	Compound^a^	Odour note	Fraction^b^	DB-5	FFAP	FD
1	Acetoin	Buttery	NBF	nd	1275	4
2	Acetic acid	Vinegar-like	AF	635	1450	4
3	2-Methylbutanal	Malty	NBF	663	912	8
4	2-Methylpyrazine	Popcorn-like	NBF	820	nd	8
5	Butanoic acid	Sweaty, cheesy	AF	835	1619	4
6	2,5-Dimethylpyrazine	Nutty	NBF	906	nd	16
7	2-Furanmethanethiol	Coffee-like	AF	907	1428	32
8	2-Acetyl-1-pyrroline	Popcorn-like	NBF	922	1371	256
9	Benzaldehyde	Almond-like	NBF	963	1524	8
10	1-Octen-3-one	Mushroom-like	NBF	977	1295	4
11	2-Acetylthiazole	Roasty, earthy	NBF	1020	1624	64
12	2-Acetylpyridine	Popcorn-like	NBF	1031	1551	16
13	4-Hydroxy-2,5-dimethyl-3(2H) furanone	Caramel-like	AF	1067	2029	8
14	2-Methoxyphenol	Smoky, sweet	NBF	1088	1858	32
15	2-Acetyl-2-thiazoline	Popcorn	NBF	1091	1755	4
16	3-Hydroxy-4,5-dimethyl-2(5H) furanone	Seasoning-like	AF	1107	2200	32
17	β-Iso-methyl ionone	Floral	NBF	1534	nd	4
18	Tetradecanal	Creamy, fishy	NBF	1601	nd	4
19	Hexadecanal	Cardboard-like	NBF	1800	nd	4
20	Octadecanal	Oily	NBF	1818	nd	4
21	Hexadecanol	Waxy, floral	NBF	1854	nd	4
22	Hexadecanoic acid	Waxy	AF	1984	nd	4

*AF* acidic fraction, *NBF* neutral and basic fraction, *FD* flavour dilution

^a^ Compounds were identified by comparing their retention indices on DB-5 and FFAP columns, mass spectra, and their aroma impressions were compared with the respective reference compounds

^b^ Fractions in which the odorants were detected by GC–O after fractionation

In addition, the presence of the coffee-like 2-furanmethanethiol and 2-acetylthiazole in the fried snail meat are of particular interest. While, majority of Sulphur compounds such as thiazoles, sulfides and thiophenes are chemically stable and can be extracted easily, thiols are very reactive and susceptible to oxidation, dimerization, and reacts with carbonyls. Hence they deserve special attention to ensure minimum losses during analysis. 2-Acetylthiazole and 2-furanmethanethiol have been reported as major odorants in coffee [26] and identified in cooked meat, popcorn and baguette bread [27]. Furthermore, 2-furanmethanethiol has been identified as a major aroma component of steamed mangrove crab (*Scylla serrata*) [28]. On the other hand, 2-acetylthiazole a product of non-enzymatic browning reactions between reducing sugars and amino acids in the presence of H_2_S [28], has been identified in nearly all cooked or roasted food aromas [28]. For instance, 2-acetylthiazole was reported as important odorant in steamed squid [21] and fried prawn meat [29].

Other thermally induced carbohydrate or protein degradation compounds such as 4-hydroxy-2,5-dimethyl-3(2H)-furanone (HDMF, furanone ^®^), and 3-hydroxy-4,5-dimethyl-2(5H)-furanone (sotolon) were detected with higher FD factors in the fried and boiled snail meats respectively (Tables 1, 2). Furanone and sotolon are important aroma compounds and are considered key flavor odorants in many food products. They are also highly appreciated in the food industry. Furanone and sotolon are products of the Maillard reaction and numerous methods for their synthesis have been published [30, 31].

Additionally, the identification of β-ionone and β-iso-methyl ionone for the first time in the snail meat was of interest. Although these aroma compounds exhibited low FD factors in the snail samples, they are known for their significant contribution to the aroma of flowers and foods [32]. In nature, β-ionone an example of Apo carotenoid is obtained by specific cleavage of β-carotenoid. This reaction is often catalyzed by the action of carotenoid cleavage deoxygenase 1 (CCD 1), which cleaves carotenoids at the 9, 10 position and 9′, 10′ position in the presence of oxygen [33]. However, Baldermann et al. [34] have shown that β-ionone can also be produced through carotenoid-cleavage like enzymes in *Enteromorpha compressa* (L.) Nees. Thus, the formation of this compound by carotenoid-cleavage like enzymes in raw snail meat seems likely.

### Contribution of aroma compounds to the overall aroma quality of the raw and thermally processed snail meats

Finally, to have an idea of the contribution of the odorants to the aroma characteristics of the raw and thermally processed snail meats exhibited in Fig. 3, the 13 odorants detected through AEDA as the key odorants (FD factors ≥ 8) (Table 3) were quantified. Results of the aroma potencies showed that fried snail meat exhibited greater potency for 3-hydroxy-4,5-dimethyl-2(5H)-furanone (sotolon), 2-acetyl-1-pyrroline, 2-furanmethanethiol and 2-methylbutanal as revealed by their high odor activity values (OAVs) (Table 3). Again, boiled snail meat exhibited similar but lower potency for the same aroma compounds as those of the fried snail meat. Moreover, the raw snail showed stronger potencies for 1-octen-3-one, β-ionone and octanal respectively. While, the OAVs indicated that 4-hydroxy-2,5-dimethyl-3(2H)-furanone (furanone), octanal, 1-octen-3-one, 2-acetylpyridine, 2-methoxyphenol and 2-methylpyrazine contributed to the seasoning, popcorn and coffee-like aroma of the thermally processed African giant snail meat. 1-Octen-3-one, octanal and β-ionone were the major contributors to the mushroom, sweaty/cheesy notes of the raw snail meat. A detailed analysis on aroma recombination experiments will be needed to determine the contribution of single odorant to the overall aroma of the snail meat.

**Table 3:** Concentrations (µg Kg^−1^ fresh weight) and odour activity values (OAVs) of aroma-active odorants (FD ≥ 8) in raw, boiled and fried giant snail (*A. fulica*)

No.	DB-5	Compound	Snail	Conc.	Snail Conc.	Odour thresholds in water µg Kg^−1^	OAVs		
			Raw	Boiled	Fried		Raw	Boiled	Fried
1	663	2-Methylbutanal	nd	16.8 ± 1.0	30.0 ± 1.0	1^a^	nd	16	30
2	820	2-Methylpyrazine	nd	100.3 ± 0.7	126.7 ± 0.4	60^b^	nd	1.7	2.1
3	906	2,5-Dimethylpyrazine	nd	40.0 ± 1.4	45.1 ± 1.5	800^b^	nd	< 1	< 1
4	907	2-Furanmethanethiol	nd	nd	4.7 ± 0.1	0.005^b^	nd	nd	940
5	922	2-Acetyl-1-pyrroline	nd	nd	123.6 ± 2.2	0.1^a^	nd	nd	1236
6	963	Benzaldehyde	13.5 ± 0.0	16.4 ± 0.1	20.0 ± 0.1	350^a^	< 1	< 1	< 1
7	977	1-Octen-3-one	1.2 ± 0.0	0.9 ± 0.0	0.1 ± 0.0	0.005^a^	240	180	20
8	1006	Octanal	45.9 ± 1.0	63.2 ± 1.0	78.9 ± 2.5	8^a^	5.7	7.9	9.9
9	1020	2-Acetylthiazole	nd	5.7 ± 0.1	15.9 ± 0.1	10^a^	nd	< 1	1.5
10	1031	2-Acetylpyridine	nd	70.0 ± 2.1	102 ± 1.5	19^a^	nd	3.7	5.4
11	1067	4-Hydroxy-2,5-dimethyl-3(2H) furanone	nd	nd	46.5 ± 1.0	5^a^	nd	nd	9.3
12	1088	2-Methoxyphenol	nd	nd	12.9 ± 0.1	2.5^a^	nd	nd	5.2
13	1107	3-Hydroxy-4,5-dimethyl-2(5H) furanone	nd	6.1 ± 0.1	11.2 ± 1.4	0.001^a^	nd	6100	11,200
14	1457	β-Ionone	5.4 ± 0.1	0.7 ± 0.0	nd	0.03^c^	180	23	nd
15	1534	β-Iso-methyl ionone	12.2 ± 0.1	1.1 ± 0.0	0.9 ± 0.0	nd	nd	nd	nd

Mean ± SD

*OAVs* odour activity value was calculated by dividing the concentration with the threshold value of compound in water, *nd* not determined

^a^Rychlik et al. [35]

^b^Tressl [36]

^c^Silva et al. [37]

### Sensory evaluation

To corroborate the analytical data, sensory evaluations were performed on the snail samples by trained panelists. Sensory evaluation of the raw, boiled and fried snail meats revealed distinct aroma characteristics (Fig. 3). While the raw snail meat exhibited sweaty/cheesy, mushroom and faint-fatty notes, the boiled snail meat was characterized by popcorn, seasoning-like, malty and mushroom notes. The fried snail elicited similar but stronger aroma notes as the boiled snail meat. In addition, the fried snail meat also had strong coffee-like nuance.

## Conclusion

Applications of the AEDA, GC–O and OAVs revealed significant differences in the aroma profiles of the raw and thermally processed snail meats. Whilst the aroma profile of the raw snail meat was dominated with the floral-like β-ionone and β-iso-methyl ionone, sweaty/cheesy-like butanoic acid, and the mushroom-like 1-octen-3-one, the boiled and fried samples were dominated with the thermally generated odorants like 2-methylpyrazine, 2,5-dimethylpyrazine, 2-acetylthiazole and 2-acetylpyridine. Among aroma-active compounds detected in the fried snail and not in the boiled snail were; 2-furanmethanethiol, 4-hydroxy-2,5-dimethyl-3(2H)-furanone, 2-methoxyphenol, 2-acetyl-2-thiazoline and some saturated long chain aldehydes. In addition, results have shown that sotolon, 2-acetyl-1-pyrroline, 2-furanmethanethiol, 2-methylbutanal, 1-octen-3-one, octanal, furanone, 2-methoxyphenol, 2-acetylpyridine, 2-acetylthiazole, and 2-methylpyrazine contributed to the overall aroma of the thermally processed snail meat. Finally, a detailed analysis on aroma recombination experiments will be needed to determine the contribution of single odorant to the overall aroma of the snail meat.

## Materials and methods

### Materials

Thirty adult giant snails (*A. marginata and A. achatina*) weighing between 82.10 and 96.40 g were collected after rainfall from three different gardens located in Port Klang, Malaysia. The shells of the snails were removed and the soft body was washed with distilled water and subsequently frozen (− 20 °C).

### Thermal processing

Thawed snail meats were divided into three batches of 200 g each. A batch was cooked in unsalted boiling water (100 °C) [20] for 15 min. After boiling, the snail was frozen with liquid nitrogen and ground into powder. A second batch was pan-fried at 160 °C without using fat as described earlier by Mall and Schieberle [20]. The frying protocol was carried out in an open pan heated with cooking gas as is done in domestic uses. The frying was continued for 8 min. The snail meat was stirred and reversed every minute for uniform cooking. After frying, the snail was cooled and frozen with liquid nitrogen before milling into powder. The third batch was used as the control.

### Chemicals

The following reference compounds: acetic acid, acetoin, 2-methylbutanal, 2-methylpyrazine, 2,5-dimethylpyrazine, 2-furanmethanethiol, 2-acetyl-1-pyrroline, benzaldehyde, 1-octen-3-one, octanal, linalool, 2-acetylthiazole, 2-acetylpyridine, 4-hydroxy-2,5-dimethyl-3(2H)-furanone, 2-methoxyphenol, 3-hydroxy-4,5-dimethyl-2(5H)-furanone, hexadecanol, octadecanal, 2.3-pentanedione, butanoic acid, β-iso-methyl ionone, β-ionone, were from Sigma-Aldrich (St. Louis MO). Stock standard solutions 10^3^ or 10^4^ µg mL^−1^ of each compound was prepared as described earlier [21].

### Sample preparation

Powdered snail meat (100 g) was blended with anhydrous sodium sulphate (50 g) and diethyl ether (300 mL) followed by continuous stirring (2 h). The obtained mixture was filtered and subjected to solvent assisted flavor evaporation (SAFE) [22]. The obtained distillate was dried over anhydrous sodium sulphate and concentrated to approximately 50 mL [23].

### Fractionation of volatiles

The SAFE distillate was treated with 150 mL of aqueous sodium bicarbonate (0.5 mol L^−1^) to yield an organic and aqueous layer respectively. The organic layer was washed twice with 75 mL of brine and dried over anhydrous sodium sulphate to produce the neutral/basic fraction (NBF). The aqueous layers were combined and acidified (pH 2.5) with HCl (16%) and extracted with diethyl ether (200 mL). The extract was subsequently dried over anhydrous sodium sulphate to yield the acidic fraction (AF). Both NBF and AF were concentrated to 100 µL each as described by Lasekan et al. [23] the resulted fractions were subjected to GC–O and GC–MS.

### Extraction of raw snail meat

Minced raw snail (200 g) was extracted as described for the thermally processed samples above. The obtained mixture was subjected to SAFE distillation [22] and extracted with dichloromethane (2 × 200 mL). The extract was dried over anhydrous sodium sulphate and the organic phase was subsequently concentrated as described above. The concentrated extract was subjected to GC–O and GC–MS.

### GC–MS and GC-FID analyses

A Shimadzu (Kyoto, Japan) QP-5050A GC–MS equipped with a GC-17 A Ver.3, a flame ionization detector (FID) and fitted differently with columns DB-FFAP and DB-5 (each, 30 m × 0.32 mm i.d., film thickness 0.25 µm; Scientific, Inc., Ringoes, NJ) was employed [24]. The gas chromatographic and mass spectrometric conditions were the same as described previously by Lasekan and Ng [26]. The HP Chemstation Software was employed for the data acquisition and mass spectra were identified using the NIST/NB575K database.

### GC–O analysis

A Trace Ultra 1300 gas chromatograph (Thermo Scientific, Waltham, MA, USA) fitted with either a DB-FFAP or DB-5 column 1:(30 m × 0.32 mm i.d., film thickness, 0.25 µm, Scientific Instrument Services, Inc., Ringoes, NJ) and an ODP 3 olfactory Detector Port (Gerstel, Mulheim, Germany), with additional supply of humidified purge air, was operated as earlier reported by Lasekan et al. [21]. The split ratio between the sniffing port and the FID detector was 1:1. Two replicate samples were sniffed by three trained panelists who presented normalized responses, with strong agreement with one another.

### Identification and quantification

The linear retention indices were calculated according to Kovats method using a mixture of normal paraffin C_6_–C_28_ as external references [24]. The identification of compounds was as described earlier by Lasekan [24]. Quantitative data were obtained by relating the peak area of each compound to that of the corresponding external standard and were expressed as µg kg^−1^.

### Aroma extracts dilution analysis (AEDA)

The extracts of snail meat were diluted step wise twofold with dichloromethane by volume to obtain dilutions of 1:2, 1:4, 1:8, 1:16 and so on [24]. Each of the obtained dilution was injected into the GC–O. The highest dilution in which an aroma compound was observed is referred to as the flavor dilution (FD) factor of that compound [25].

### Aroma profile analysis

Snail meats (raw, boiled and fried) (40 g each) were placed inside glass container (7 cm × 3.5 cm) and were orthonasally analyzed as described by Lasekan and Ng [26]. Reference compounds were: 3-hydroxy-4,5-dimethyl-2(5H)-furanone (seasoning), 2-acetyl-1-pyrroline (popcorn), 2-methylbutanal (malty), 2-furfurylthiol (coffee-like), benzaldehyde (almond), 1-octen-3-one (mushroom), butanoic acid (sweaty/cheesy), and linalool (floral). An unstructured scale was used to rate each descriptor by panelists. The scale was from 0 to 10, where 0 = not detectable, 5 = weak, and 10 = strong. Final results were produced as a web plot.
